# Extreme environmental tolerance and space survivability of the moss, *Physcomitrium patens*

**DOI:** 10.1016/j.isci.2025.113827

**Published:** 2025-11-20

**Authors:** Chang-hyun Maeng, Yuji Hiwatashi, Keita Nakamura, Osamu Matsuda, Hajime Mita, Kaori Tomita-Yokotani, Shin-ichi Yokobori, Akihiko Yamagishi, Atsushi Kume, Tomomichi Fujita

**Affiliations:** 1 Graduate School of Life Science, Hokkaido University, Sapporo, Japan; 2 Faculty of Science, Hokkaido University, Sapporo, Japan; 3 School of Food Industrial Sciences, Miyagi University, Sendai, Japan; 4 Graduate School of Food, Agricultural and Environmental Sciences, Miyagi University, Sendai, Japan; 5 Faculty of Science, Kyushu University, Fukuoka, Japan; 6 Department of Life, Environment, and Applied Chemistry, Fukuoka Institute of Technology, Fukuoka, Japan; 7 Faculty of Life and Environmental Sciences, University of Tsukuba, Tsukuba, Japan; 8 School of Life Sciences, Tokyo University of Pharmacy and Life Sciences, Hachioji, Japan; 9 Faculty of Agriculture, Kyushu University, Fukuoka, Japan

**Keywords:** biological sciences, plant biochemistry, plant biology, plant development, plant physiology, plants, space sciences

## Abstract

Climate change highlights the importance of understanding life’s survival limits for addressing global challenges and supporting future human habitation beyond Earth. Plants, as photosynthetic organisms, play a vital role in sustaining life. Bryophytes, such as mosses, show notable extremotolerance, but despite studies on environmental responses in the model moss *Physcomitrium patens*, its survival under extreme conditions in space remains unclear. We tested protonemata, brood cells, and spores encased in sporangium under simulated space environments, identifying spores as the most resilient, and subsequently exposed them to the space environment outside the International Space Station. After 9 months in space, over 80% of the spores survived, retaining their ability to germinate. These results demonstrate the remarkable resilience of *P. patens* spores in space and reveal the potential of terrestrial plants to endure extreme environments, providing insight into bryophyte adaptation and offering a foundation for future applications in space exploration and extraterrestrial habitats.

## Highlights

• *P. patens* spores survive simulated space conditions with high resilience
• Over 80% of spores remained viable after 9 months outside the ISS
• Sporangium may provide protection against UV, heat, and intensive light stresses
• Bryophyte could be used for planetary greening and life support systems

## Graphical abstract

**Figure:**
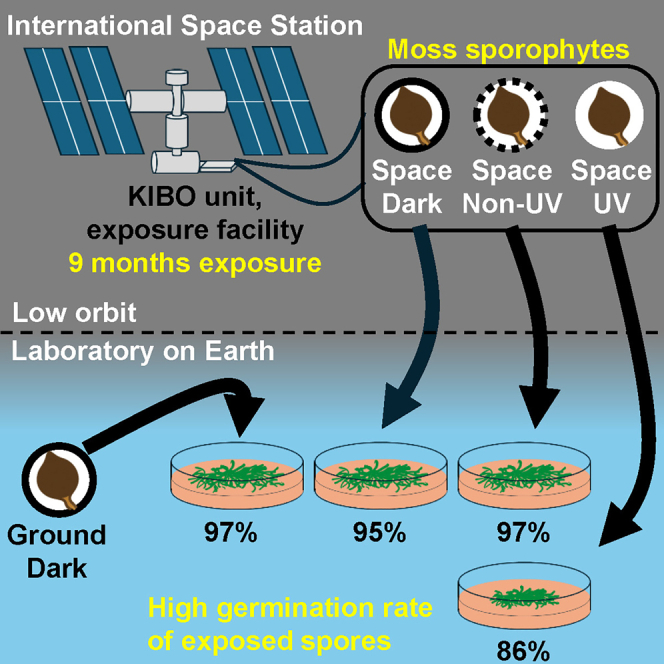

## Introduction

As Earth’s environment undergoes rapid changes in recent years, it has become increasingly important to explore new possibilities for the survival of life beyond our planet. Understanding the resilience of Earth-born organisms in extreme and unfamiliar conditions, such as the space environment, is a crucial step toward expanding human habitats other than Earth like the Moon or Mars.^1^ Studying the survival limits of living organisms in both terrestrial and space environments will not only enhance our understanding of their adaptability but also help us prepare for the challenges of sustaining ecosystems. However, research on these survival limits is still in its early stages.

In the course of evolution, organisms have transitioned from aquatic to terrestrial environments. The terrestrial environments are harsher than aquatic environments due to factors such as desiccation, ultraviolet (UV) radiation,^2^ and temperature variability. Bryophytes were the first organisms to adapt to these terrestrial conditions,^3^ making them one of the most resilient species in terms of environmental changes and enabling them to survive to the present day. About 500 million years ago, bryophytes advanced onto the ground and formed the basis of the terrestrial ecosystem, such as increasing the oxygen level of the Earth’s atmosphere.^4^ Since then, bryophytes have survived without becoming extinct despite changes in the Earth’s environment (e.g., mass extinction in Earth’s history).

Despite the remarkable environmental resilience demonstrated by bryophytes on Earth, little is known about their ability to survive in various extreme environments on Earth or in space. Bryophytes, particularly mosses, are known for their remarkable tolerance to desiccation, freezing, and radiation, making them ideal model organisms for studying life under extreme environments. Several studies have reported survival and recovery of bryophytes after prolonged exposure to harsh conditions.^5^^,^^6^^,^^7^ In particular, some studies have reported that mosses can survive, for example, in Mars-like simulated conditions.^8^^,^^9^ It was reported that gametophytes of *Grimmia sessitana* showed a survival rate about 80% after 31 days in the Martian atmosphere and 17 days in a Martian UV-simulated environment,^8^ and remarkably, dried gametophytes of another moss, *Syntrichia caninervis*, did not show a significant decrease in the final recovery rate even after 7 days of treatment in a Martian-simulated environment (atmosphere, pressure, and temperature).^9^ These mosses are known as species particularly resistant to desiccation and low temperatures, suggesting that mosses may retain considerable resilience even in extreme space environments. Space experiments, such as the Biorisk experiment, EXPOSE-E, EXPOSE-R, and EXPOSE-R2 revealed that some vascular plants and microorganisms are capable of surviving harsh space conditions while studying how organisms respond to the space environment.^10^^,^^11^ However, most of the space exposure experiments involving plants have focused on vascular plants, such as crops, while research involving bryophytes has been very limited.^8^^,^^10^ Therefore, to investigate how long bryophytes can survive in actual space conditions, we used the model moss *Physcomitrium patens* to explore the extent of extreme environments it can withstand and to test its viability in space.

*P. patens* is recognized as a valuable model organism in molecular biology due to its structural simplicity, ease of genetic manipulation, fully sequenced genome, and unique physiological traits.^12^ *P. patens* also has been extensively studied over the past 20 to 30 years in research on evolutionary development, environmental responses, and the evolution of terrestrial plants.^13^ Previous research has revealed that its abscisic acid (ABA) response and signaling pathways, which are essential for environmental stress responses, are well conserved compared to those in other vascular plants.^14^ In environmental response studies on mosses, research has focused on protonemata and gametophores due to their ease of preparation. When exogenous ABA or environmental stress such as salt or drought is applied to the protonemata of moss, round-shaped cells are formed in response^15^ (Figure S1). The round-shaped cells, called brood cells (brachycytes), have higher stress resistance than typical protonemata and act as diaspores. After fertilization between egg and sperm, moss produces sporophytes to transition to the next generation (Figure S1). In the sporophyte, meiosis produces a large number of spores, which are enclosed by outer cell layers forming the sporangium (Figure S1). Although some studies have reported stress tolerance in the sporophyte generation,^16^ environmental responses in the sporophyte phase, compared to the haploid protonemal or gametophore phases, remain largely unknown.

To investigate differences in environmental responses across tissues of *P. patens*, we first compared and evaluated stress tolerance across different cells and tissues in the haploid and diploid phases: protonemata (juvenile filamentous cells), brood cells (stress-induced vegetative diaspores), and sporophytes (diploid reproductive structures that encase spores). In our assays, sporophytes were used as the physical unit for stress exposure, and viability was determined by the germination rate of spores released from the sporangium. The results revealed that encased spores exhibit greater stress tolerance than brood cells and protonemata. To further explore the viability of *P. patens* in space, we conducted exposure experiments using sporophytes on the exposed section of the International Space Station (ISS). Remarkably, even after 9 months of exposure to space conditions, over 80% of the encased spores germinated upon return to Earth. These findings underscore the extraordinary resilience of *P. patens* and its potential for advancing our understanding of plant survival in extreme environments, both terrestrial and extraterrestrial.

## Results

### UV, freezing, and heat tolerance of tissues of *P. patens*

**Figure 1:**
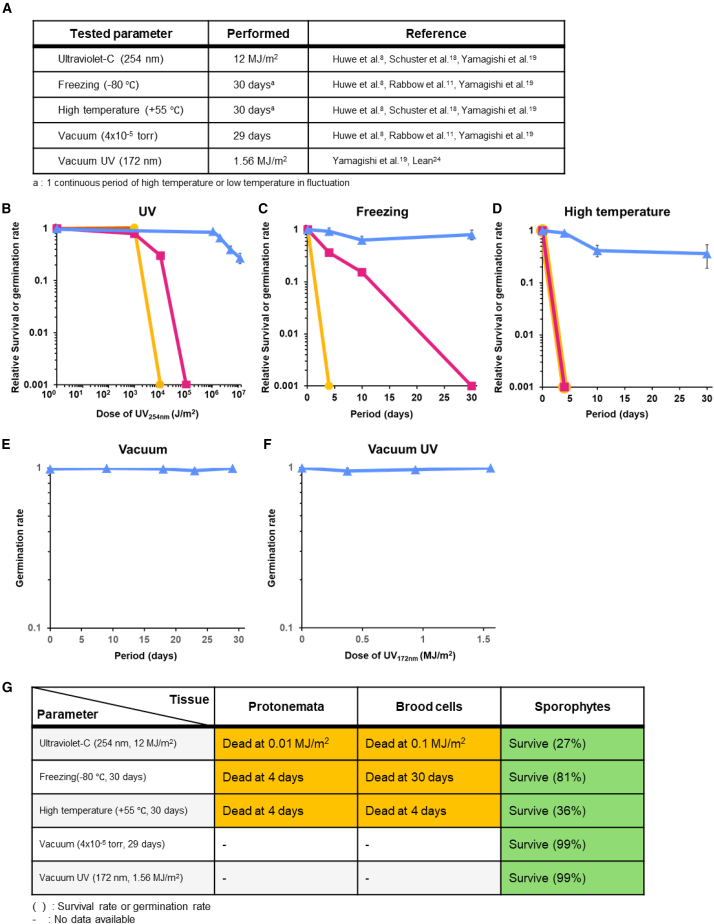
UV, freezing, heat tolerance, vacuum, and vacuum UV tolerance of *P. patens* and its usability for space exposure experiments Survival rate of protonemata (orange circle), brood cells (magenta square), and germination rate of spores (blue triangle) after treatment of stress. (A) Parameter for verification test of space experiment. (B) Protonemata and brood cells were irradiated UVC (254 nm) dose of 1 to 100 kJ/m^2^. Sporophytes were irradiated UVC dose of 1 to 12 MJ/m^2^. Protonemata and brood cells were completely dead after 10 and 100 kJ/m^2^, while germinated protonemata from spores was alive even after 12 MJ/m^2^ UVC irradiation. (C) Survival or germination rate after treatment with −80°C for 30 days. Protonemata was completely dead after 4 days of treatment. Brood cells and spores were alive, but spores showed higher viability than brood cells. (D) Survival or germination rate after treatment with 55°C for 30 days. Protonemata and brood cells showed 0% of survival rate, while spores showed 40% of germination rate. (E) Germination rate of spores after vacuum (4 × 10^−5^ torr) treatment for 9, 18, 23, and 29 days. Minimal damage was observed in every treatment period. (F) Germination rate of spores after vacuum UV (172 nm) for dose of 0.38, 0.94, and 1.56 MJ/m^2^. No significant damage was observed in all doses of VUV (*n* ≥ 3, error bar: SD). (G) Evaluation of the usability for space experiment on each parameter in different tissues. Orange indicates unusable (dead), and green indicates usable (survive). For freezing and heat treatments, a 30-day duration was selected to represent one low-temperature period and one high-temperature period in the ISS orbital cycle.

As a representative bryophyte, *P. patens* provides a valuable model for understanding how early land plants adapted to environmental stress through distinct cellular strategies. Its life cycle includes specialized tissue types with defined roles: protonemata for vegetative growth, brood cells for survival and dispersal as vegetative diaspores, and sporophytes for reproduction, contributing to genetic diversification from the parental generation.^17^ Each of these tissues likely employs unique mechanisms for stress adaptation. We examined the tolerance of *P. patens* tissues (Figure S1) to environmental stresses, including UV radiation, freezing, and high temperature, which are key factors representative of the harsh conditions encountered in space, such as the orbit of the ISS (Figure 1A). Protonemata, ABA-induced brood cells,^15^ and mature sporophytes encasing spores were subjected to these conditions. Protonemata and brood cells were assessed based on their cellular survival, while germination rates of spores extracted from the sporophytes were used to evaluate their tolerance.

UV radiation is a critical factor to consider in exposure experiments conducted outside the ISS.^8^^,^^18^^,^^19^ In particular, UVC radiation, rarely encountered naturally on Earth, is abundant in space.^20^ To evaluate UVC tolerance, we exposed three *P. patens* tissues to 254 nm UVC light. Protonemata showed no significant change at 1,000 J/m^2^ but dropped to 0% survival at 10,000 J/m^2^ (Figure 1B). Brood cells exhibited greater tolerance, with survival rates of 79% at 1,000 J/m^2^ and 30% at 10,000 J/m^2^, but none survived at 100,000 J/m^2^ (Figure 1B). In contrast, spores within sporophytes retained germination rates of 87%, 65%, 39%, and 27% at 1, 2, 5, and 12 MJ/m^2^, respectively (Figure 1B), exhibiting approximately 1,000 times greater tolerance than brood cells. These findings reveal a hierarchy of UVC tolerance, with encased spores being the most resilient, followed by brood cells and protonemata.

Freezing is a critical factor in simulating extreme conditions outside the ISS.^8^^,^^11^^,^^19^ Prolonged freezing can severely impact plant survival.^21^ To evaluate deep-freezing tolerance, *P. patens* tissues were exposed to −80°C for up to 30 days (Figure 1A). Protonemata showed complete mortality after 4 days (Figure 1C). Brood cells exhibited moderate tolerance, with survival rates of 36% and 15% after 4 and 10 days, respectively, but none survived beyond 30 days (Figure 1C). Spores demonstrated exceptional tolerance, with germination rates of 93%, 63%, and 80% after 4, 10, and 30 days, respectively (Figure 1C). Even after 30 days at −80°C, the germination rate declined by only 20%. To simulate deep space conditions,^22^ spores in sporophytes were exposed to −196°C, resulting in a 9% germination rate after 8 days (Figure S2). These findings highlight the superior freezing tolerance of *P. patens* spores encased in sporangium, likely representing an adaptive strategy to maintain dormant state until suitable germination conditions arise.

Then, protonemata, brood cells, and sporophytes were exposed to 55°C, a simulated high temperature on the ISS orbit, for up to 30 days to evaluate high temperature tolerance.^8^^,^^18^^,^^19^ Protonemata and brood cells exhibited complete mortality within 4 days (Figure 1D), indicating high sensitivity to this range of heat stress. In contrast, spores displayed significantly higher tolerance, maintaining a 90% germination rate after 4 days, 41% after 10 days, and 36% after 30 days (Figure 1D).

Our findings demonstrate that spores encased in sporangium exhibit significantly higher environmental tolerance compared to protonemata and brood cells. These results suggest that the spores of *P. patens* may possess greater intrinsic tolerance than other cell types to environmental stresses. Since only the spores survived the wide range of simulated space environments (Figure 1A), we selected mature sporophytes for further experiments.

### Vacuum and vacuum UV ray tolerance of *P. patens* spores

Evaluating tissue survival under vacuum conditions is critical for space exposure experiments. We conducted a vacuum survival test at 4 × 10^−5^ torr (approximately 5.33 × 10^−3^ Pa), replicating the low orbit conditions (Figure 1A).^8^^,^^19^ After 29 days, *P. patens* spores derived from sporophytes exhibited a 99% germination rate, indicating minimal damage and supporting their potential for long-term survival in a vacuum (Figure 1E).

Next, we investigated the combined effect of vacuum UV radiation (VUV, 100–200 nm). VUV radiation, with shorter wavelength than UVC, poses a significant hazard in space due to the absence of atmospheric shielding. To simulate this, we exposed spores encased in sporangium to a 172-nm excimer lamp.^23^ Spores in sporangium were exposed to VUV doses of 0.38, 0.94, and 1.56 MJ/m^2^, and the extracted spores retained germination rates of 99% (Figure 1F). The estimated 1-year VUV dose in ISS orbit is ∼0.5 MJ/m^2^, based on the spectrum of a 172-nm lamp.^24^ The encased spores’ ability to withstand higher doses underscores their exceptional resistance to VUV radiation. Thus, our results suggest that *P. patens* spores encased in sporangium are highly tolerant to both vacuum and VUV radiation, with the potential to survive at least 1 year of space exposure. Notably, the sporangium structure encasing the spores may serve as a protective barrier,^25^ absorbing VUV radiation and preventing it from penetrating the interior. This structural defense likely contributes to the spores’ extreme tolerance to VUV radiation.

Collectively, our findings demonstrate the robust tolerance of *P. patens* spores, encased in sporophyte structures, to VUV and UVC radiation, freezing, and high temperatures, reinforcing their potential suitability for extended space exposure experiments (Figure 1G).

### Exposure of *P. patens* sporophytes to the external environment of the ISS

Building on ground-based experiments, we aimed to expose mature sporophytes to actual space conditions. The space environment imposes multiple stress factors that are not encountered on Earth, including extreme temperature fluctuations, radiation, UV light, vacuum, and altered gravity.^10^^,^^18^

**Figure 2:**
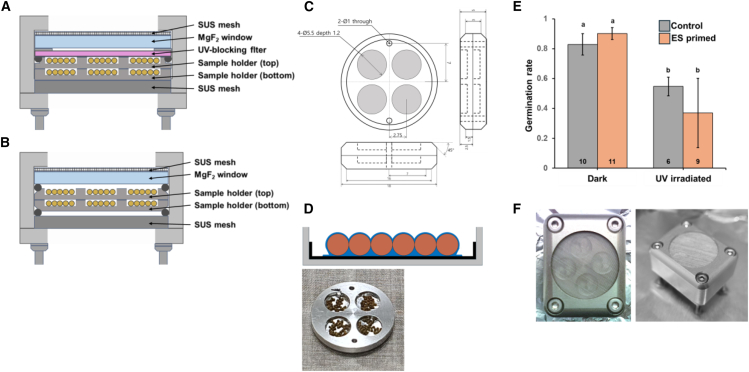
Design of the space exposure unit and assemblage of samples for the space exposure experiment (A) The space exposure unit with a UV-blocking filter installed. A UV-blocking filter, which only allows light above 400 nm to pass, was added below the MgF_2_ window. A double-layered sample holder was installed, designed so that the samples placed in the lower section would not be exposed to light. (B) The space exposure unit without a UV-blocking filter installed. (C) Design of sample holder (unit: mm). (D) Top: a schematic showing a single well (5.5 mm in diameter) with samples (sporophytes) installed. Brown: sporophytes, blue: ES of cyanobacteria as adhesive, black: aluminum foil, gray: sample holder. Bottom: the actual appearance of the sporophytes installed in the sample holder. (E) Impact assessment of ES. After treating both ES-primed sporophytes and control sporophytes with dark conditions or 2 MJ/m^2^ of UVC (254 nm), the germination rate was observed. Different letters indicate statistically significant differences as determined by ANOVA with Tukey’s HSD post hoc test. The numbers inside the bar graphs represent the sample sizes. Error bar: SD. (F) The completed space exposure unit.

Sporophytes for the space exposure experiment were prepared as shown in Figure 2. Units were equipped with MgF_2_ windows blocking wavelengths below 110 nm (Figures 2A and 2B).^19^ A custom sample holder ensured proper placement, and cyanobacterial extracellular substance (ES) was used as an adhesive to immobilize the exposure sample (Figures 2C and 2D),^26^ without exhibiting any cytotoxicity or adverse effects under UVC exposure (Figure 2E). Four experimental groups were prepared to evaluate the effects of UV radiation and other space-related factors: the laboratory control group (ground dark), space control group (space dark), space group shielded from UV with a UV-cut filter (space non-UV), and space UV group without a UV-cut filter (space UV).

The exposure unit was launched aboard Cygnus NG-17 and placed on the ISS exposure facility from March 4, 2022, to December 23, 2022, excluding an onboard storage period from July 13 to July 26, 2022. This resulted in 283 days of space exposure. The unit was returned to Earth via SpaceX CRS-16 on January 11, 2023. Upon receipt, spores extracted from sporangium were sown on agar medium to assess germination.

**Figure 3:**
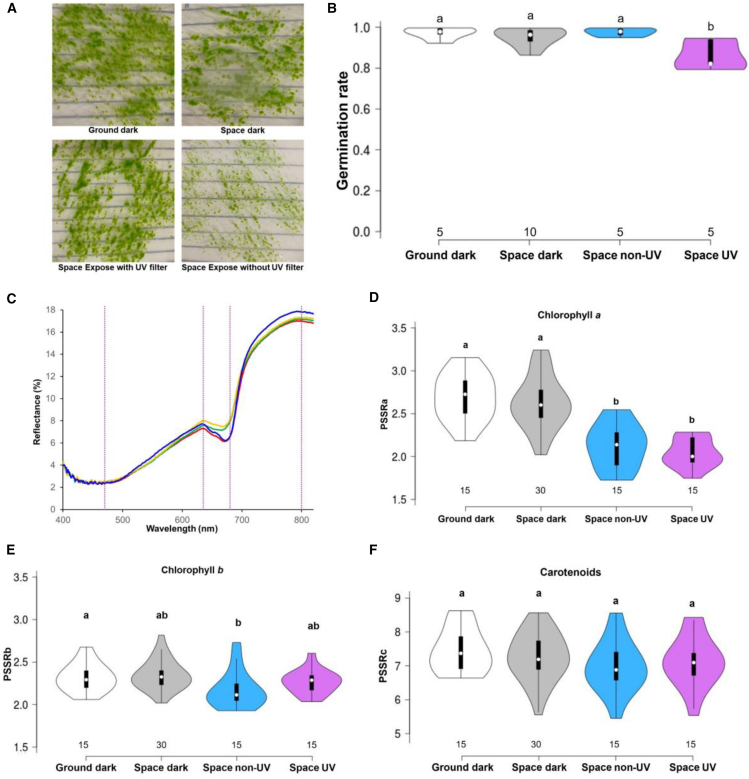
Germination rate of spores and pigment change of sporophytes after Tanpopo4 flight (A) Protonemata grown by spore germination on BCDAT (10 mM Ca^2+^) medium after Tanpopo4 flight. Scale bars: 1 cm. (B) Germination rate after space exposure experiment. Ground dark: laboratory dark condition; Space dark: space dark condition (sporophytes in bottom sample holder); Space non-UV: space light condition (sporophytes in top sample holder with UV-blocking filter); Space UV: space light condition (sporophytes in top sample holder without UV-blocking filter). (C) Reflectance on the surface of the sporophyte. The average reflectance was measured from 15 to 30 sporophytes. Blue: ground dark; red: space dark; orange: space expose with filter; green: space expose without filter. The purple vertical dashed lines indicate the wavelengths used for PSSR calculations, representing 470, 635, 680, and 800 nm from left to right. (D–F) Comparison of chlorophyll *a*, chlorophyll *b*, and carotenoids using PSSRa, PSSRb, and PSSRc calculated from reflectance spectra. Different letters indicate statistically significant differences as determined by ANOVA with Tukey’s HSD post hoc test. The numbers above the *x* axis represent the sample size.

As shown in Figure 3A, spores germinated and protonemata grew in all groups. Germination rates in the ground dark (97%) and space dark (95%) groups were comparable, indicating limited effect from vacuum, temperature fluctuations, or microgravity. The space non-UV group showed a 97% germination rate, suggesting that visible and infrared light had negligible effects (Figure 3B). However, the space UV group exhibited a germination rate of 86%, an 11% reduction, highlighting UV radiation as a significant detrimental factor (Figure 3B). We evaluated only the germination rates of protonemata derived from space-exposed spores, without analyzing later developmental stages or detailed protonemal morphology. Potential abnormalities in filament development, sexual organ formation, anatomical structures, or physiological features were not examined and remain to be investigated in future studies.

A linear regression model based on germination decline in the space UV group produced the formula Y = 0.9714 × e^−4E−4X^ (Y: germination rate, X: exposure days). The decimal reduction dose (D_10_; dose required to kill 90% of spores) suggests that spores encased in sporophytes could survive up to 5,600 days, approximately 15 years, under space conditions (Figure S3). These findings underscore the remarkable resilience of *P. patens* spores, protected by sporangium, to prolonged space exposure. However, this estimation is based only on two points (initial and 9 months post exposure) and should therefore be interpreted with caution due to the limited data supporting such extrapolation.

### Pigment changes on sporophyte surface

Chlorophyll and carotenoids absorb light, but excessive exposure can lead to pigment degradation.^27^^,^^28^ Given the critical importance of pigments like chlorophyll in photosynthesis and their potential contribution to oxygen production in space, we investigated these pigment changes following space exposure. Importantly, pigments were measured directly from exposed sporophytes, rather than from regenerated protonemal tissues after germination. To enable this direct assessment, we employed a reflectance-based method across the visible to near-infrared spectrum,^29^ instead of conventional pigment extraction techniques.^30^ This non-invasive approach allows rapid and direct evaluation of pigment integrity in response to space conditions and is particularly advantageous when working with limited returned samples (e.g., ∼8 mg per 100 sporophytes, 120 sporophytes per group in our space experiments).

In the reflectance-based method, pigment-specific simple ratios (PSSRs) were employed to estimate chlorophyll *a*, chlorophyll *b*, and carotenoid levels.^29^^,^^31^ However, since PSSR was not originally developed for use with moss sporangial tissues, we evaluated whether these indices reliably reflect pigment composition in the sporangium. To this end, we conducted validation experiments comparing the conventional pigment extraction method followed by absorbance measurements^30^ with PSSR values. Pigments were extracted from sporangia in the ground dark group, and their absorbance spectra were measured. Absorption peaks corresponding to chlorophyll *a* (∼680 nm) and chlorophyll *b* (∼640 nm) were detected, along with notably high absorbance in the blue region (400–460 nm), suggesting the presence of yellow pigments, such as carotenoids, at high concentrations (Figure S4A). Subsequently, we obtained the reflectance spectra of sporophytes in the ground dark group using hyperspectral imaging. Mean reflectance values at 470, 635, 680, and 800 nm were calculated from 15 individual sporangia (Figure S4B).

For chlorophyll *a*, the values obtained from the extraction and PSSR methods were 55.5 and 63.0 mg/m^2^, respectively (Figure S4C). For chlorophyll *b*, the corresponding values were 32.4 and 35.3 mg/m^2^, respectively (Figure S4C). These results indicate good agreement between extraction-based measurements and PSSR estimates (PSSRa and PSSRb), supporting the consistency of chlorophyll quantification across methods. In contrast, carotenoid content showed a greater discrepancy, with values of 11.6 mg/m^2^ from the extraction method and 40.9 mg/m^2^ from the PSSR method (Figure S4C), suggesting that carotenoids or other pigments not eluted with a solvent, *N,N*-dimethylformamide, may be present in the PSSRc wavelength range (470 nm).

Based on this validation of the PSSR indices, we then compared pigment changes across samples. As shown in Figures 3D–3F, chlorophyll *a* (PSSRa) levels showed no significant difference between the ground dark and space dark control groups. However, space-exposed samples (space non-UV and space UV) exhibited a 20% reduction in PSSRa (Figure 3D). This reduction persisted even in the space non-UV group, suggesting that degradation was driven by visible and infrared light rather than UV radiation. In contrast, chlorophyll *b* (PSSRb) and carotenoids (PSSRc) exhibited minimal changes, with no significant differences among the space-exposed conditions (space dark, space non-UV, and space UV) (Figures 3E and 3F). These findings suggest that chlorophyll *a* is particularly susceptible to the light environment in space, highlighting its potential utility as a sensitive indicator of photodamage under extraterrestrial conditions.

## Discussion

To the best of our knowledge, this is the first report demonstrating the survival of bryophytes following exposure to space and subsequent return to the ground. Our study confirms that *P. patens* spores in sporangium exhibit remarkable tolerance to extreme environments, both under simulated conditions in ground laboratory and in low earth orbit environments outside the ISS. Notably, 9 months of exposure to temperature fluctuations, vacuum, radiation, and microgravity in space had no significant effect on germination rates; no differences were observed among the ground dark, space dark, and the space non-UV groups (Figure 3B), suggesting that space-related environmental factors, excluding UV radiation, exert a limited effect on spore survival. A significant reduction in germination was observed in the space UV group, identifying UV radiation as a primary stressor (Figure 3B). However, the 86% germination rate indicates that *P. patens* spores encased in sporangium can potentially survive over 9 months in space, even under UV exposure.

The exceptional resilience of *P. patens* encased spores may result from a synergistic effect between the inherent stress resistance of the spores and the protective role of the sporangium, which likely functions as a stress-resistant reproductive structure.^32^ Acting as a structural barrier, the sporangium shields spores from challenging terrestrial conditions such as desiccation, UV exposure, and temperature extremes, both physically and chemically, until environmental conditions become favorable.^25^^,^^33^ This dual strategy, resilience at the spore level combined with external protection by the sporangium, may represent an early evolutionary adaptation in bryophytes for terrestrial survival, which may have been subsequently co-opted into the analogous protective function of the seed coats in vascular plants, and potentially relevant to survival in space environments.

In UVC irradiation experiments, *P. patens* protonemata showed complete lethality at 10,000 J/m^2^, while 30% of ABA-induced brood cells survived at this dose (Figure 1B). While exogenous ABA has been shown to enhance UVB resistance in vascular plants,^34^ little is known about UVC tolerance in *P. patens* brood cells or ABA’s role in mediating this response in land plants. Our results demonstrate that ABA-inducible brood cells possess enhanced UVC tolerance, likely facilitated by antioxidants such as bisbibenzyls, flavonoids, and carotenoids, which may absorb UVC radiation.^35^^,^^36^^,^^37^ These findings provide valuable insights into UVC tolerance mechanisms, a critical consideration for space environments devoid of atmospheric filtering.

To place these findings in a broader biological context and given that UVC is one of the major environmental stressors in space, we compared UVC tolerance across a wide range of taxa (Table 1). Notably, *P. patens* spores exhibited exceptional resistance, with a 50% lethal dose (LD_50_) of 3.9 MJ/m^2^ and a D_10_ of 24.5 MJ/m^2^ (calculated from Figure 1B, Y = 0.894e^−1E−7X^). Likewise, the gametophytes of the desert moss *S. caninervis* demonstrated remarkable tolerance, with complete survival after 7 days of UVC exposure at 1.6 W/m^2^ (∼0.97 MJ/m^2^),^9^ suggesting that the spores of this species may exhibit even greater UVC tolerance. These values far exceed those reported for *Deinococcus radiodurans*, one of the most radio-resistant bacteria (D_10_ of a few kJ/m^2^),^38^ and are also greater than those observed in seeds of some angiosperms, such as sunflower (*Helianthus annuus*, LD_50_ of several tens of kJ/m^2^).^39^ However, certain angiosperm seeds, including those of *Arabidopsis thaliana* and *Nicotiana tabacum*, exhibited even greater tolerance than *P. patens* spores.^40^

**Table 1:** Comparison of UVC tolerance of different species

Groups	Species (tissues)	UV dose (kJ/m^2^)	Survival	Reference
Mosses	*Physcomitrium patens* (Hedw.) Bruch & Schimp. (protonemata)	10	lethal	this study
Mosses	*Physcomitrium patens* (Hedw.) Bruch & Schimp. (brood cells)	10	30%	this study
Mosses	*Physcomitrium patens* (Hedw.) Bruch & Schimp. (spores)	11,800	27%	this study
Mosses	*Grimmia sessitana* De Not. (gametophytes)	10	21%	Huwe et al.^8^
Mosses	*Syntrichia caninervis* Mitt. (gametophytes, dried)	967	100%	Li et al.^9^
Seed plants	*Arabidopsis thaliana* (L.) Heynh. (seedlings)	10	35%	Otake et al.^41^
Seed plants	*Arabidopsis thaliana* (L.) Heynh. (seeds)	60,000	60%	Tepfer et al.^40^
Seed plants	*Nicotiana tabacum* L. (seeds)	60,000	50%	Tepfer et al.^40^
Seed plants	*Glycine max* (L.) Merr. (seeds)	41.4	92%	Araújo et al.^42^
Seed plants	*Convolvulus arvensis* L. (seeds)	60,000	100%	Tepfer et al.^40^
Seed plants	*Triticum aestivum* L. (seeds)	43.2	96%	Foroughbakhch Pournavab et al.^39^
Seed plants	*Helianthus annuus* L. (seeds)	43.2	72%	Foroughbakhch Pournavab et al.^39^
Seed plants	*Pinus maximartinezii* Rzed. (seeds)	1.1	36%	Foroughbakhch Pournavab et al.^39^
Green alga	*Chlamydomonas reinhardtii* P.A.Dangeard (logarithmic phase cells)	0.3	5%	Nandita et al.^43^
Cyanobacterium	*Nostoc* sp. (akinete cells)	1.2	10%	Tomita-Yokotani et al.^44^
Fungi	*Aspergillus niger* Tiegh. (spores)	1	10%	Cortesão et al.^45^
Fungi	*Schizosaccharomyces pombe* Lindner, 1893 (growing phase cells)	0.2	10%	Yasuhira et al.^46^
Bacteria	*Bacillus subtilis* (Ehrenberg, 1835) Cohn, 1872 (spores)	0.2	10%	Slieman et al.^47^
Bacteria	*Deinococcus radiodurans* Brooks & Murray, 1981 (early stationary phase cells)	1	10%	Pogoda de la Vega et al.^38^
Animals	*Caenorhabditis elegans* (Maupas, 1900) (individuals, hermaphrodite)	0.06	lethal	Murakami et al.^48^
Animals	*Hypsibius dujardini* (Doyère, 1840) (individuals, hydrated)	2.5	lethal	Horikawa et al.^49^
Animals	*Ramazzottius varieornatus* Bertolani & Kinchin, 1993 (individuals, hydrated)	10	lethal	Horikawa et al.^49^
Animals	*Polypedilum vanderplanki* Hinton, 1951 (individuals, dried larvae)	80	30%	Ryabova et al.^50^
Animals	*Mus musculus* Linnaeus, 1758 (mouse embryonic fibroblasts cells)	0.075	20%	Begović et al.^51^
Animals	*Homo sapiens* Linnaeus, 1758 (human epidermal skin cells [HaCaT)	0.08	11%	Chang et al.^52^

In contrast, desiccation-tolerant animals such as tardigrades (*Hypsibius dujardini* and *Ramazzottius varieornatus*) and UV-resistant insects’ hydrated larvae undergoing anhydrobiosis (*Polypedilum vanderplanki*) failed to match the UVC tolerance observed in *P. patens* spores.^49^^,^^50^ Similarly, spores of bacteria and fungi, such as *Bacillus subtilis* and *Aspergillus niger*, showed only limited UVC resistance. Thus, these patterns highlight that certain plant structures, namely spores and seeds, tend to exhibit superior UV resistance, likely due to the presence of specialized UV-screening pigments such as flavonoids and carotenoids, which help protect DNA and cellular structures from UV-induced damage.^53^^,^^54^ While microorganisms lack these plant-derived pigments, they may utilize functionally similar protective compounds. For instance, dipicolinic acid found in *B. subtilis* spores is known to confer protection against UV-A and UV-B radiation and may also contribute to UVC resistance.^47^

The observed contrast in UVC resistance between plants and animals may stem from fundamentally different survival strategies; plants are evolutionarily adapted to passively withstand harsh environments, whereas animals typically avoid stress through mobility. Whether the high UVC tolerance found in moss spores and in the seeds of seed plants is primarily pigment-driven or also involves other protective mechanisms, such as stress-protective sugars like trehalose, which contributes to drought tolerance in plants and has been reported to enhance UV resistance in animal cells by scavenging reactive oxygen species (ROS),^55^^,^^56^ remains an open question. Future comparative studies on the biochemical and structural bases of UV resistance across diverse taxa will provide insights into both conserved and lineage-specific mechanisms. Such knowledge will be invaluable for selecting biological systems suitable for extraterrestrial use and for developing robust bioregenerative life support systems (BLSSs).

Prolonged freezing at −80°C for up to 30 days revealed significantly higher tolerance in spores compared to brood cells (Figure 1C). However, spores’ tolerance may still lag behind that of seeds from vascular plants. Several studies revealed extreme freezing tolerance in vascular plant seeds accomplished during cryopreservation; for example, *Prunus avium* L. seeds exhibit 37% of germination after 1 year at −196°C,^57^ and *Fagus crenata* seeds achieved 50% germination after 6 months at −196°C.^58^ In contrast, *P. patens* spores derived from sporangium showed only 9% of germination after 8 days at −196°C (Figure S2). Interestingly, the moss *S. caninervis* gametophores showed exceptional freezing tolerance, surviving −80°C for 5 years and −196°C for 30 days,^9^ highlighting that the remarkable freezing tolerance capabilities exist in some mosses.

Heat tolerance was similarly highest in spores derived from sporangium compared to protonemata and brood cells in *P. patens* (Figure 1D). The sporangium consists of only a few cell layers, making it unlikely to act as a heat insulator capable of protecting internal spores from prolonged high-temperature exposure. This suggested that spores themselves have high-temperature tolerance, although, as far as we know, there is no report to examine such high temperature tolerance on spores in bryophytes. Alternatively, our data raised the question: what mechanism enables sporangium to shield spores from such high-temperature stress? Given the structural similarity between sporophytes, where spores are encased by the sporangium, and seeds, where embryos are protected by the seed coat, we hypothesized that the sporangium may provide heat tolerance akin to the seed coat. However, while studies have examined the effects of high temperatures on seed germination and biochemical responses,^59^^,^^60^ the specific mechanisms underlying heat tolerance in seeds—and by extension, in sporophytes—remain poorly understood. This underscores the need to further explore the adaptive mechanisms of bryophyte spores to thermal stress.

Photosynthetic pigments, particularly chlorophylls, are essential for light absorption and energy conversion. Chlorophyll *a*, residing in the photosystem reaction center, is indispensable for photosynthesis. Our results revealed a significant reduction in chlorophyll *a* in space-exposed samples (Figure 3D). It should be noted that the observed decrease in chlorophyll *a* was detected mainly in the desiccated cell layers of the sporangium and thus reflects physical degradation rather than a physiological acclimation to the space environment. Chlorophyll *a* is susceptible to photodegradation because it is readily oxidized by thylakoid-associated peroxidative activity.^61^ It is also reported that chlorophyll *a* is a target for ROS generated during prolonged exposure to intense light conditions such as visible and infrared in space.^62^ Various stresses in space, including intense light condition, cosmic radiation, desiccation, and temperature, can increase ROS levels,^63^^,^^64^^,^^65^ and these ROS may have contributed to the degradation of chlorophyll *a.*^66^ This effect may in fact be manifested by the significantly greater intensity of direct sunlight on the ISS than on Earth, due to the absence of atmospheric scattering.^67^^,^^68^ Consequently, plant tissues in orbit are exposed to levels of solar radiation that terrestrial plants have never experienced. These findings highlight the need to consider long-term stability of chlorophyll *a* when designing BLSSs, as prolonged exposure may exacerbate these effects and compromise overall ecosystem function.

As humanity endeavors to expand habitation into space, developing BLSSs is essential for sustaining our life beyond Earth. Systems such as BIOS-3 and Biosphere 2^69^^,^^70^ have explored plant-based solutions for oxygen generation and carbon dioxide removal. While electrolysis remains the primary method for oxygen production,^71^ BLSS research increasingly focuses on self-sustaining ecosystems. Bryophytes present a promising alternative to algae and crops due to their efficient carbon fixation, oxygen production, and adaptability to low-light conditions.^72^ As pioneer plants, bryophytes have the potential to transform regolith into fertile soil, facilitating ecosystem development on other planets, similar to peat moss improving soil fertility on Earth.^4^^,^^73^ Our study highlights the resilience of *P. patens* spores to space environments and offers insights into survival mechanisms that could inform future space exploration and extraterrestrial ecosystem development.

### Limitations of the study

This study focused on a single model moss, *P. patens*, and thus may not capture the diversity of responses across other bryophytes or plants. The molecular mechanisms underlying sporangium resistance remain unclear, including whether they parallel those of seeds or extend to other bryophytes and vascular plants. Space exposure was limited to 9 months aboard the ISS, and outcomes may differ with longer durations. Chlorophyll *a* degradation was observed, but insufficient resolution for chlorophyll *b* and limited sample numbers preclude firm conclusions about changes in the a/b ratio or selective breakdown. Furthermore, the light intensity that induced chlorophyll *a* loss was not quantified, and additional factors such as vacuum and temperature may also have contributed. These limitations underscore the need for longer-term, comparative, and mechanistic studies.

## Resource availability

### Lead contact

Further information and requests for resources and reagents should be directed to and will be fulfilled by the lead contact, Tomomichi Fujita (tfujita@sci.hokudai.ac.jp).

### Materials availability

This study did not generate new unique reagents. Cyanobacterial ESs extracted from *Nostoc* sp. HK-01, used as an adhesive, are available from the lead contact upon reasonable request. The *P. patens* strains used in this study are available from the lead contact upon request.

### Data and code availability

• All raw data and hyperspectral image data reported in this paper will be shared by the lead contact upon request.
• Any additional information required to reanalyze the data reported in this paper is available from the lead contact upon request.

## Acknowledgments

We would like to express our gratitude to Mr. Yasutaka Sasaki of the Technical Division at the Faculty of Science, Hokkaido University, for fabricating the sample holder. We also extend our thanks to Ms. Hirono Kobari for cultivating the sporophytes, Ms. Sayaka Takahashi (Fukuoka Institute of Technology) for VUV experiment, and Dr. Ong (Tsukuba University) for providing the cyanobacteria ES. We would like to thank the Tanpopo team (Dr. Tomoko Abe, Dr. Hiroshi Katoh, Dr. Yoshitaka Bessho, Dr. Hirofumi Hashimoto, Dr. Hajime Yano, Dr. Kensei Kobayashi, and Dr. Shunta Kimura) for their hard work in ensuring the smooth progress of the experiment. This work was supported by DX scholarship 10.13039/501100005946Hokkaido University (JST SPRING, grant no. JPMJSP2119) to C.-h.M. T.F. was funded by 10.13039/501100001691JSPS KAKENHI grants JP23K17399, JP21K19272, and JP25H01374. This work was also supported by the Astrobiology Center of National Institutes of Natural Sciences (AB032001 and AB042003).

## Author contributions

Conceptualization, A.K. and T.F.; methodology, C.-h.M., Y.H., K.N., O.M., H.M., K.T.-Y., S.-i.Y., A.Y., A.K., and T.F.; validation, C.-h.M. and T.F.; investigation, C.-h.M.; writing – original draft, C.-h.M. and T.F.; writing – review and editing, C.-h.M., Y.H., K.N., O.M., H.M., K.T.-Y., S.-i.Y., A.Y., A.K., and T.F.; supervision, T.F.; funding acquisition, H.M., K.T.-Y., S.-i.Y., and T.F.

## Declaration of interests

The authors declare no competing interests.

## STAR★Methods

### Key resources table

**Table:** 

REAGENT or RESOURCE	SOURCE	IDENTIFIER
**Biological samples**		

Cyanobacterial extracellular substances (ES) from *Nostoc* sp. HK-01	Ong et al.^26^	N/A

**Chemicals, peptides, and recombinant proteins**		

Abscisic acid (2-cis,4-trans-Abscisic acid)	Sigma-Aldrich	Cat#862169

**Experimental models: Organisms/strains**		

*Physcomitrella patens* (Hedw.) Bruch & Schimp subsp. *Patens*	Nishiyama et al.^74^	N/A

**Software and algorithms**		

Adobe Photoshop 2023	Adobe Inc.	https://www.adobe.com/products/photoshop.html; RRID:SCR_014199
RStudio (version 2023.06.1 + 524)	Posit, PBC	https://posit.co/; RRID:SCR_000432
R (version 4.3.1)	R Core Team	https://www.r-project.org/; RRID:SCR_001905
Visual Studio Community 2022	Microsoft	https://visualstudio.microsoft.com/
Math.NET Numerics (v5.0.0)	Math.NET	https://numerics.mathdotnet.com/

**Other**		

Peat pellet	Jiffy Products International BV	Jiffy-7
Incubator	Yamato Scientific Co., Ltd.	IC43
Ultra-low temperature freezer	PHCbi	MDF-C8V1-PJ
UV germicidal lamp GL-15	HotaluX Co., Ltd. (Japan)	GL-15
VUV Excimer lamp (172 nm)	Ushio Inc. (Japan)	UER20H-172V
Fluorescence microscope	Leica Microsystems	DMLB
Filter set A (for fluorescence microscope)	Leica Microsystems	Excitation: 34 BP0–380 nm; Emission: LP 425 nm
Short-wavelength cut-off filter (UV 400 nm)	Asahi spectra	LUX400
MgF_2_ window	Yamagishi et al.^19^	N/A
Tanpopo exposure unit	Yamagishi et al.^19^	N/A
Tanpopo4 sample holder	This paper	N/A
Hyperspectral camera	Themis Vision Systems Inc.	VNIR-200R
Closed-circuit television lens	Schneider-Kreuznach Company	21-010425 Xenoplan 23 mm f/1.4
Motorized shutter device	Sutter Instrument Company	Lambda SC + IQ35-SA SmartShutter
Anhydrous synthetic quartz-transmission lights	NIPPON P·I Co., Ltd.	PDL-S-250VAB
DC halogen lamps with aluminum reflector	NIPPON P·I Co., Ltd.	PLL-250/AL
Stepping motor-driven single axis robot	MISUMI Corp.	RS206-C1-N-1-800
CCD detector	Sony Corp.	ICV285AL
Polytetrafluoroethylene (PTFE) filter paper	Toyo Roshi Kaisha, Ltd.	PF100
UV-Vis spectrophotometer	Hitachi	U-3010

### Experimental model and study participant details

#### Plant materials and growth conditions

*Physcomitrium patens* (formerly known as *Physcomitrella patens*) subsp *patens* collected from Gransden Woods (Ashton and Cove, 1977) was utilized for the preparation of protonemata, brood cells, and sporophytes. To prepare protonemata, *P. patens* tissues were homogenized using a Polytron homogenizer and subsequently subcultured in BCDAT agar medium (1 mM MgSO_4_·7H_2_O, 1.84 mM KH_2_PO_4_, 10 mM KNO_3_, 0.045 mM FeSO_4_·7H_2_O, 5 mM (NH_4_)_2_C_4_H_4_O_6_, 0.22 μM CuSO_4_·5H_2_O, 10 μM H_3_BO_3_, 0.23 μM CoCl_2_·6H_2_O, 0.1 μM Na_2_MoO_4_·2H_2_O, 0.19 μM ZnSO_4_·7H_2_O, 2 μM MnCl_2_·4H_2_O, 0.17 μM KI, 1 mM CaCl_2_·2H_2_O, 0.8% Agar)^74^ with 0.1% (v/v) DMSO for the mock control. Brood cells were induced by subculturing in BCDAT agar medium supplemented with 50 μM abscisic acid.^75^ Both protonemata and brood cells were cultured for two days after subculture in a growth chamber (Sanyo, MLR-350) set at 25°C under continuous fluorescent light (Toshiba, FHF32EX-W-H) with an intensity of 60 μmol photons/m^2^s. The light intensity was measured using photo-radiometer (Deltaohm, HD2102.2) equipped with a PAR probe (Deltaohm, LP471PAR).

To prepare sporophytes, protonemata vegetatively propagated on BCDAT medium were transplanted onto sterile peat pellet (Jiffy Products International BV, Jiffy-7) in a plastic box. The peat pellets were half-immersed in sterile water and incubated in a growth chamber Nippon Medical & Chemical Instruments, LH-200-RD) under continuous white light at an intensity of 60 μmol photons/m^2^s at 25 °C for a month to produce gametophores. Subsequently, the peat pellets containing gametophores were moved to another growth chamber (Panasonic Healthcare, MLR-352H-PJ) and cultured under 8-h light and 16-h dark conditions at an intensity of 40 μmol photons/m^2^s at 15 °C for at least 7 weeks to generate sporophytes. Mature sporophytes were collected using tweezers under stereomicroscope and dried at room temperature. These sporophytes were then stored at 4 °C until further use. Only sporophytes with a diameter of at least 300 μm were selected for the experiments.

### Method details

#### Stress treatments

For UVC irradiation, 2-day-old *P. patens* protonemata or brood cells were exposed to a UV germicidal lamp (HotaluX, Ltd., GL-15 germicidal lamp) at an intensity of 25 W/m^2^. The UVC doses (1, 10, and 100 kJ/m^2^) were controlled by adjusting the exposure time. The intensity of UVC was measured using a photo-radiometer (Deltaohm, HD2102.2) equipped with a UVC probe (Deltaohm, LP471UVC). After irradiation, the samples were incubated in a growth chamber at 25 °C under continuous fluorescent light for 7 days, and survival rates were subsequently evaluated. Fully dried sporophytes were affixed to plastic Petri dishes using double-sided tape to prevent movement due to their spherical shape and then irradiated with UVC at the same intensity (25 W/m^2^) and doses of 1, 3, 5, and 12 MJ/m^2^. Germination rates were assessed immediately following treatment by sowing the spores.

Freezing treatments were conducted using a −80 °C deep freezer (PHCbi, MDF-C8V1-PJ). Protonemata and brood cells were kept on the BCDAT agar medium in the deep freezer for 4 days and 10 days, respectively. After freezing, the samples were transferred to a fresh medium and incubated in a growth chamber at 25 °C under continuous fluorescent light for 7 days to assess survival rates. Sporophytes were placed in 1.5 mL tubes and placed in the deep freezer for 4, 10, and 30 days, after which they were immediately sown to assess germination rates. In all freezing experiments, humidity inside the deep freezer was maintained below 15%RH.

Heat treatment was performed using an incubator (Yamato Scientific, IC43) set to 55 °C. Protonemata and brood cells were incubated on the BCDAT agar medium at 55 °C for 4 days. After heat treatment, the samples were transferred to a fresh medium and incubated in a growth chamber at 25 °C under continuous fluorescent light for 7 days to evaluate survival rates. Sporophytes were placed in 1.5 mL tubes and incubated in the incubator for 4, 10, and 30 days. Immediately after heat treatment, the sporophytes were sown to assess germination rates. In all heat treatment experiments, humidity inside the incubator was maintained below 10%RH.

Vacuum and vacuum ultraviolet (VUV) treatments were applied to sporophytes. For vacuum treatment, sporophytes were placed in stainless-steel mesh cube (self-made, 10 mm per side, made from 200 mesh stainless steel) and subjected to a vacuum of 4 × 10^−5^ torr for 9, 18, 23, and 29 days. After treatment, sporophytes were sown to evaluate germination rates. VUV treatment was conducted using a 172 nm excimer lamp (Ushio, UER20H-172V) under a vacuum of several tens of torr, with doses of 0.38, 0.94, and 1.56 MJ/m^2^.

All experiments were conducted with a sample size of at least three replicates.

#### Evaluation of survival and germination rates

The survival rate of protonemata and brood cells were evaluated using image analysis. After 7 days of incubation following stress treatment, protonemata and brood cells were observed under a fluorescence microscope (Leica Microsystems, DBLM) equipped with filter set A (Leica Microsystems, Excitation: 34 BP0–380 nm; Emission: LP 425 nm), and both bright-field and UVA fluorescence images were captured. In bright-field images, areas appearing green were considered as living cells and brown or transparent cells were considered dead. In UVA fluorescence images, cells exhibiting red chlorophyll autofluorescence were regarded as living cells and cells exhibiting blue fluorescence were considered as dead cells. Survival rates were calculated by analyzing the proportion of area of living cells relative to the total area. Survival rates were calculated by analyzing the proportion of the area occupied by living cells relative to the total area, using Adobe Photoshop 2023 (Adobe Inc.).

For spores extracted from sporangium, germination rates were assessed by direct sowing. Stress treated sporophytes were placed in 1 mL of sterile water in a 1.5 mL tube. Sporangium was disrupted using sterile tweezers to extract spores until the brown sporangia turned translucent, indicating sufficient release of spores. Spore suspension was sown on BCDAT agar medium with 10 mM CaCl_2_·2H_2_O. The entire spore suspension was applied onto the medium surface in multiple drops, with each drop containing approximately 80 μL. The media in which spores were sown were incubated under white light (60 μmol photons/m^2^s) at 25 °C until complete germination. If no change in germination was observed compared to previous observations, germination was considered complete, and the germination rate at that point was defined as the final germination rate.

#### Space exposure

To isolate the effects of UV radiation, two types of space exposure units were prepared. The exposure unit was constructed based on that used in previous Tanpopo missions.^19^ The first unit was equipped with a UV-blocking filter (Asahi spectra, LUX400) allowing only visible light and infrared radiation to pass through (Figure 2A). The second unit allowed full exposure to visible light, infrared radiation, and UV radiation, including UVA, UVB, and UVC (Figure 2B). All units are equipped with windows made of MgF_2_ capable of blocking wavelengths below 110 nm.^69^^,^^70^ The sample holder was redesigned to match the size and quantity of sporophytes and was fabricated from A2017 duralumin at the Technical Division of Hokkaido University. Both units were fitted with sample holders containing *P. patens* sporophytes, designed with a dual-layer structure to ensure complete shielding from light in specified conditions (Figure 2A and 2B). Each sample holder contains four wells with a diameter of 5.5 mm (Figure 2C). We placed 30 *P. patens* sporophytes in each well (total 120 sporophytes per each group) and used cyanobacterial extracellular substance (ES) extracted from *Nostoc* sp. HK-01 as an adhesive to secure sporophytes, preventing movement or dispersal (Figure 2D). Cyanobacterial ES was selected as the adhesive due to use of cyanobacteria in the previous Tanpopo missions.^26^ The dried ES was resuspended in sterile water to a final concentration of 4% (w/v). After applying 5 μL of ES solution to each well, a piece of aluminum foil was placed over it and dried for 30 min to secure the aluminum foil to the well. An additional 5 μL of ES solution was then applied onto the aluminum foil surface, and sporophytes were placed on top. The setup was dried again for 30 min to firmly secure the sporophytes to the aluminum foil. The exposure unit was stored with a desiccant until just before the space exposure experiment.

As a part of the Tanpopo 4 mission, the assembled exposure units (Figure 2F) were mounted on the Exposed Experimental Bracket Attached on i-SEEP (ExBAS). ExBAS was placed on Exposed Facility (EF) of Japanese Experiment Module (JEM) “KIBO” of ISS for 9 months, a total of 283 days from March 2022 to December 2022.

#### Hyperspectral imaging

Hyperspectral images of sporophytes were captured using an imaging system composed of a push-broom visible (VIS) to near-infrared (NIR) hyperspectral camera (VNIR-200R, Themis Vision Systems Inc., USA), a closed-circuit television (CCTV) lens (Schneider-Kreuznach Company, 21-010425 Xenoplan 23 mm f/1.4), and a motorized shutter device (Sutter Instrument Company, Lambda SC + IQ35-SA SmartShutter) attached in front of the lens. Illumination was supplied through a facing pair of anhydrous synthetic quartz-transmission lights (NIPPON P·I, PDL-S-250VAB) placed adjacent to DC halogen lamps with aluminum reflector (NIPPON P·I, PLL-250/AL). A sample tray was transferred by a stepping motor-driven single axis robot (MISUMI, RS206-C1-N-1-800). The camera was equipped with a 12 μm-wide slit and a silicon charge-coupled-device (CCD) detector (Sony, ICV285AL), having sensitivity in the wavelength range of 400–980 nm, and was capable of acquiring up to 1,392 pixel-wide and 1,040 spectral-band line image at a single exposure (with 1 × 1 binning mode). In this study, we applied 2 × 2 binning mode, affording 696 × 520 horizontal and spectral resolution. As the pixel size of the CCD was 6.45 × 6.45 μm and the horizontal field-of-view (FOV) was set to 114 mm by applying a working distance of 292 mm, the resulting images had spatial resolution of ca. 155 ppi with a spectral sampling pitch of 1.29 nm. Brightness of the images was calibrated to absolute reflectance by linear interpolation using an image of a sheet of white polytetrafluoroethylene (PTFE) filter paper (Toyo Roshi Kaisha, PF100) as the 90% reflectance standard and a darkfield image (0% reflectance).

#### Pigment extraction and spectrophotometric analysis

Ten mature sporophytes from the ground dark group were selected and transferred into a 1.5 mL microcentrifuge tube containing 50 μL of sterile water. Using sterilized tweezers, the sporophytes were gently crushed to release spores. Only the sporangia were isolated and placed on tissue paper to remove surface water. The air-dried sporangia were then transferred into a new 1.5 mL tube containing 100 μL of DMF. The sporangia were thoroughly ground using sterile tweezers, and the tube was incubated at 4 °C for 24 h in the dark.

The liquid portion of the sporangia extract was collected and subjected to spectrophotometric analysis using a UV-Vis spectrophotometer (Hitachi, U-3010). Absorbance was measured by performing a wavelength scan from 400 to 800 nm, with data recorded at 1 nm intervals. DMF was used as the blank for all measurements.

#### Calculation of PSSR

The PSSR was calculated as follows:

PSSRa = R_800_/ R_680_ for chlorophyll *a*,

PSSRb = R_800_ / R_635_ for chlorophyll *b*,

PSSRc = R_800_ / R_470_ for carotenoids,

where R_800_, R_680_, R_635_, and R_470_ represent the reflectance at each wavelength.^29^

Reflectance values were obtained from hyperspectral images, and PSSR values were calculated using Microsoft Excel for Microsoft 365 (Microsoft).

### Quantification and statistical analysis

Software for operating the hyperspectral imaging system, and for analyzing hyperspectral images was developed in managed C# code using Microsoft Visual Studio Community edition (version 2022, https://visualstudio.microsoft.com/) with an extension of Math.NET Numerics library (version 5.0.0, https://numerics.mathdotnet.com/).

All ANOVA tests were performed with Tukey’s HSD posthoc using R (version 4.3.1) in RStudio (version 2023.06.1 + 524, https://posit.co/).
