# Supplementary material for: Extreme environmental tolerance and space survivability of the moss, Physcomitrium patens
Source: iScience. 2025 Nov 20;28(12):113827. doi: 10.1016/j.isci.2025.113827 (PMC12767186; doi:10.1016/j.isci.2025.113827)
Supplement: Document S1. Figures S1–S4 [file mmc1.pdf]

iScience, Volume *28*

Supplemental information

Extreme environmental tolerance and space survivability of the moss, *Physcomitrium patens*

Chang-hyun Maeng, Yuji Hiwatashi, Keita Nakamura, Osamu Matsuda, Hajime Mita, Kaori Tomita-Yokotani, Shin-ichi Yokobori, Akihiko Yamagishi, Atsushi Kume, and Tomomichi Fujita

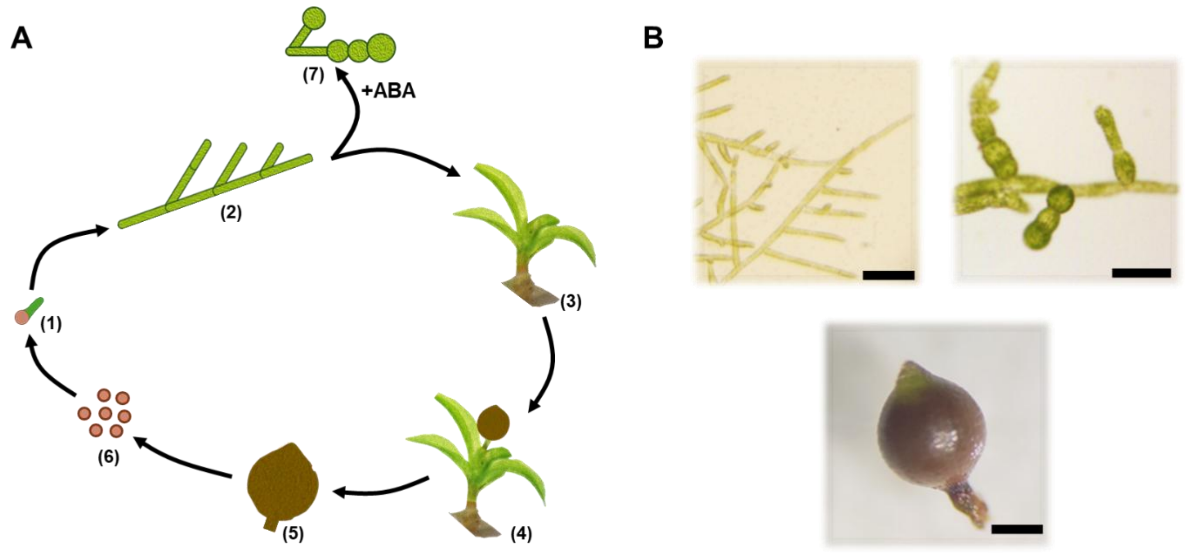

Supplementary Figure 1. Tissues of *P. patens* used in this study

(A) Life cycle of *P. patens*. *P. patens* uses spores as reproductive cells. (1) Spores germinate in an appropriate environment. (2) Germinated spores grow to form filament-shaped juvenile cells called protonemata. (3) Over time, an adult gametophore is formed, and (4) under appropriate temperature and light conditions, a sporophyte to create spores is formed at the tip of the gametophore. (5) Thousands of spores are contained within the mature sporophyte, which is enclosed by several outer cell layers that form the sporangium, and (6) the spores are released by physical forces. (7) If protonemata get environmental stress or is treated with exogenous abscisic acid (ABA), brood cells, which are round-shaped protonemata, are formed. (B) In this study, protonemata (upper left), brood cells (upper right), and sporophytes (bottom) were used among the tissues of *P. patens*. (Scale bar: 100, 50, 200 μm respectively)

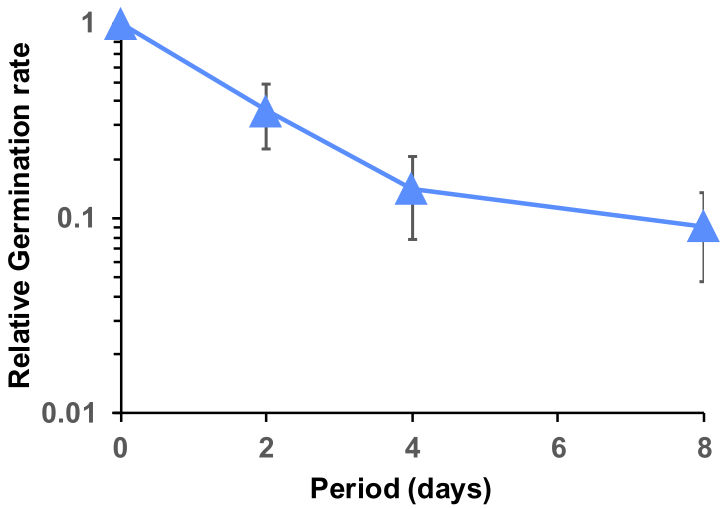

Supplementary Figure 2. Germination rate after -196 °C freezing treatment

Survival or germination rate after treatment with liquid nitrogen (-196°C) for 2, 4, 8 days. Sporophytes were placed in cryotubes and placed in liquid nitrogen in a cryopreservation chamber. Immediately after 2, 4, and 8 days of cryogenic treatment, each cryotube was left at 4°C for 30 min and then seeded in BCDAT (10 mM Ca^{2+}) medium. The media in which spores were sown were incubated under white light (60 μmol photons/m^2s) at 25°C until complete germination. (n≥5, error bar: S.D.)

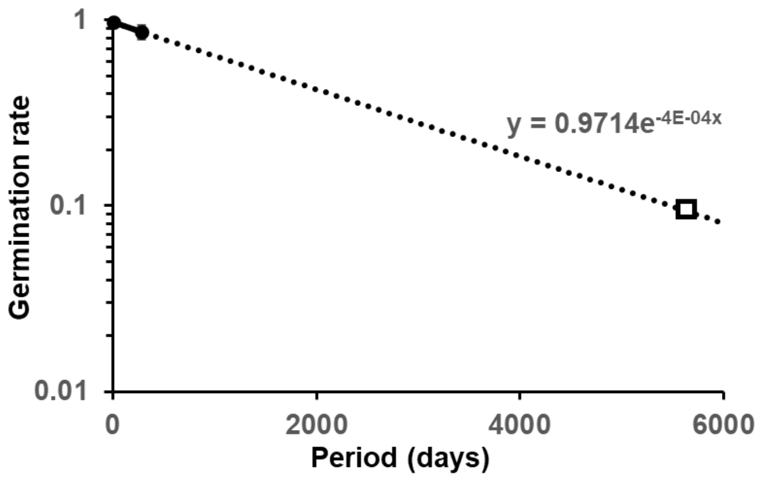

Supplementary Figure 3. Prediction of germination rate

Germination rates observed after 283 days of space exposure were used to predict the results of longer space exposure. The solid line represents the germination rate observed during the Tanpopo 4 mission. The dotted line shows the decreasing trend of germination rate calculated through exponential decay model. The open squares represent the predicted D_{10}. Error bar: S.D.

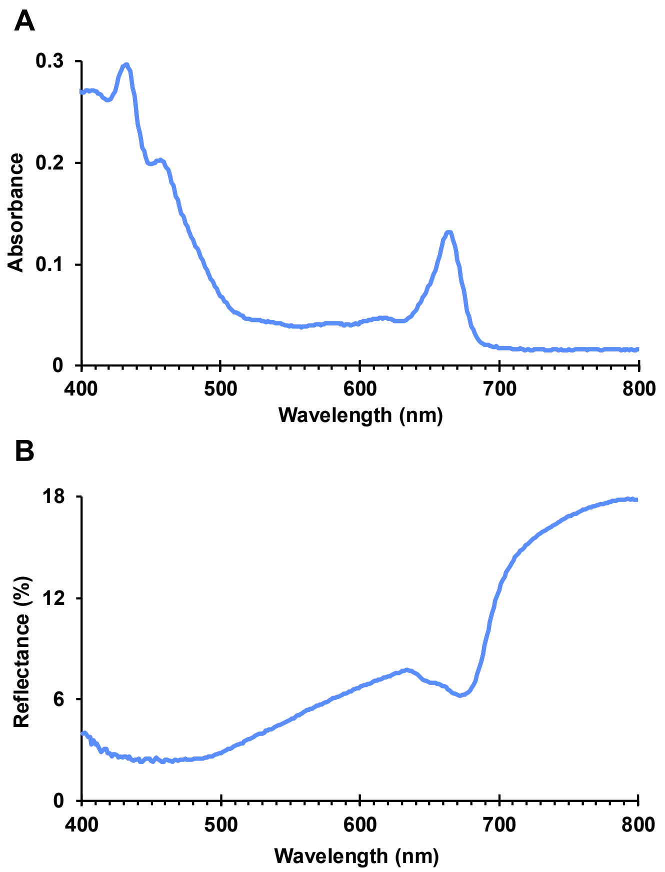

C

Pigment	Derived from	Concentration (mg/m²)	Reference
Chlorophyll *a*	Absorbance	55.5	Wellburn[30]
	PSSR	63.0	Blackburn[29]
Chlorophyll *b*	Absorbance	32.4	Wellburn[30]
	PSSR	35.3	Blackburn[29]
Carotenoids	Absorbance	11.6	Wellburn[30]
	PSSR	40.9	Blackburn[31]

Supplementary Figure 4. Validation of PSSR Method against Extraction Method

(A) Absorption spectrum of the extract obtained from sporangia of ground dark group. (B) Reflectance spectrum of the ground dark group obtained through hyperspectral imaging. (C) Comparison between pigment concentrations calculated from average PSSR and those calculated from the absorbance of the extract. The quantification of each pigment based on absorbance was performed in triplicate.
